# Overview of the actions to combat bacterial resistance in large hospitals*

**DOI:** 10.1590/1518-8345.3952.3407

**Published:** 2021-04-12

**Authors:** Mariana Sanches de Mello, Adriana Cristina Oliveira

**Affiliations:** 1 Universidade Federal de Minas Gerais, Escola de Enfermagem, Belo Horizonte, MG, Brazil.; 2 Hospital Socor, Belo Horizonte, Minas Gerais, Brazil.

**Keywords:** Cross Infection, Drug Resistance, Bacterial, Patient Safety, Epidemiological Monitoring, Hospitals, Health Personnel, Infecção Hospitalar, Resistência Bacteriana a Antibióticos, Segurança do Paciente, Vigilância Epidemiológica, Hospitais, Pessoal de Saúde, Infección Hospitalaria, Farmacorresistencia Bacteriana, Seguridad del Paciente, Monitoreo Epidemiológico, Hospitales, Personal de Salud

## Abstract

**Objective:**

to analyze, in the clinical practice of large hospitals, how the adoption of measures to prevent and control the spread of bacterial resistance has occurred, and to propose a score for the institutions’ adherence.

**Method:**

a cross-sectional study carried out in 30 large hospitals of Minas Gerais, from February 2018 to April 2019, after approval by the Ethics and Research Committee. Interviews were conducted with hospital managers, with Hospital Infection Control Services coordinators, and with the care coordinators of the Inpatient Units and Intensive Care Center. In addition, observations were made of the adoption of preventive measures by the multidisciplinary team in the care units.

**Results:**

in the 30 participating hospitals, 93.3% (N=28) had protocols for prophylactic antibiotics, and 86.7% (N=26) performed their audit, 86.7% (N=26) for therapeutic antibiotics and 83.3% (N=25) their audit; 93.3% (N=56) used gloves and cloaks for patients in contact precautions, and 78.3% (N=47) of the professionals were unaware of or answered incompletely on the five moments for hand hygiene. In the score to identify the adoption of measures to control bacterial resistance, 83.3% (N=25) of the hospitals were classified as partially compliant, 13.3% (N=04) as deficient, and 3.4% (N=01) as non-adoption.

**Conclusion:**

it was found that the recommended measures to contain bacterial resistance are not consolidated in the clinical practice of the hospitals.

## Abstract (pt)

**Objetivo:**

analisar, na prática clínica dos hospitais de grande porte como tem ocorrido a adoção das medidas de prevenção e controle da disseminação da resistência bacteriana e propor um escore de adesão das instituições.

**Método:**

estudo transversal realizado em 30 hospitais de grande porte de Minas Gerais, no período de fevereiro de 2018 a abril de 2019, após aprovação pelo Comitê de Ética e Pesquisa. Realizaram-se entrevistas com os gestores dos hospitais, com os coordenadores dos Serviços de Controle de Infecção Hospitalar e com os coordenadores assistenciais das Unidades de Internação e Centro de Terapia Intensiva. Além disso, conduziram-se observações da adoção das medidas de prevenção pela equipe multiprofissional nas unidades assistenciais.

**Resultados:**

nos 30 hospitais participantes, 93,3% (N=28) apresentaram protocolos para antibióticos profiláticos, e 86,7% (N=26) realizavam sua auditoria, 86,7% (N=26) para antibióticos terapêuticos e 83,3% (N=25) sua auditoria; 93,3% (N=56) utilizavam luvas e capotes para pacientes em precaução de contato, e 78,3% (N=47) dos profissionais desconheciam ou responderam de forma incompleta sobre os cinco momentos para higienização das mãos. No escore para identificar a adoção das medidas de controle da resistência bacteriana, 83,3% (N=25) dos hospitais foram classificados como com adesão parcial, 13,3% (N=04) com adesão deficiente e 3,4% (N=01) como não adoção.

**Conclusão:**

constatou-se que as medidas recomendadas para contenção da resistência bacteriana não estão consolidadas na prática clínica dos hospitais.

## Abstract (es)

**Objetivo:**

analizar, en la práctica clínica de los hospitales de gran porte, cómo se ha producido la adopción de medidas preventivas y de control de la propagación de resistencias bacterianas y proponer un puntaje de adhesión de las instituciones.

**Método:**

estudio transversal realizado en 30 hospitales de gran porte de Minas Gerais, de febrero de 2018 a abril de 2019, previa aprobación del Comité de Ética e Investigación. Se realizaron entrevistas con los administradores de los hospitales, con los coordinadores de los Servicios de Control de Infección Hospitalaria y con los coordinadores de atención de las Unidades de Internación y del Centro de Cuidados Intensivos. Además, se hicieron observaciones sobre la adopción de medidas preventivas por parte del equipo multidisciplinario en las unidades de atención.

**Resultados:**

en los 30 hospitales participantes, el 93,3% (N=28) presentaban protocolos de antibióticos profilácticos, y el 86,7% (N=26) realizaron su auditoría, el 86,7% (N=26) de antibióticos terapéuticos y el 83,3% (N=25) realizaron su auditoría; El 93,3% (N=56) utilizó guantes y batas para los pacientes en precaución de contacto, y el 78,3% (N=47) de los profesionales desconocía o respondía de forma incompleta sobre los cinco momentos de higiene de manos. En el puntaje para identificar la adopción de medidas para el control de la resistencia bacteriana, el 83,3% (N=25) de los hospitales se clasificaron como con adhesión parcial, el 13,3% (N=04) con adhesión deficiente y el 3,4% (N=01) como sin adopción.

**Conclusión:**

se descubrió que las medidas recomendadas para contener la resistencia bacteriana no están consolidadas en la práctica clínica de los hospitales.

## Introduction

Health care-related infections (HAIs) are defined by the National Healthcare Safety Network (NHSN) as systemic or localized conditions resulting from the action of infectious agents or their toxins, and can manifest themselves after 72 hours of admission or after the patient›s discharge^(^ 1 ^)^. It is estimated that 70% of the HAIs are associated with antibiotic-resistant bacteria as the causative agent^(^ 2 ^)^.

In a global context, bacterial resistance has direct implications for patient safety. It prolongs their stay in the hospital, increases the chances of hospital readmission, the use of extended-spectrum antibiotics and the risk of death, mainly due to the absence of therapeutic alternatives^(^ 3 ^-^ 7 ^)^.

Bacterial resistance can be considered an epidemic with severe consequences. According to the author’s projection, starting in 2050, bacterial resistance will be responsible for the death of nearly ten million patients each year, surpassing the current number of deaths from cancer and other diseases^(^ 8 ^)^. In addition, a high percentage of potentially lost years of life was estimated due to infections related to resistant bacteria in the European Union, reinforcing the issue as a worldwide public health problem^(^ 9 ^)^.

Bacterial resistance associated with HAIs occurs in all patient care units. Despite being a concern of all services, it has been more frequently registered in patients in Intensive Care Units (ICUs). ICUs are identified as the epicenter of antibiotic-resistant bacteria, with an overall higher incidence rate than that of the rest of inpatient units in health care institutions^(^ 10 ^-^ 11 ^)^.

In this sense, three main pillars for the prevention and control of bacterial resistance are pointed out: improved adherence to hand hygiene, standard and isolation precautions, and rational use of antibiotics^(^ 4 ^,^ 12 ^-^ 13 ^)^.

Although such measures are widely recognized as effective in reducing HAIs and, consequently, in the spread of resistant microorganisms, numerous studies point to a low knowledge of the measures for their adoption among the health professionals^(^ 14 ^-^ 17 ^)^.

Given the above, it was proposed to answer the following question: How has the adoption of preventive and control measures for the spread of bacterial resistance in the clinical practice of large hospitals in the state of Minas Gerais? It is also clear that the proposal is in line with the strategy of the World Health Organization (WHO) and of the National Plan of the National Health Surveillance Agency (*Agência Nacional de Vigilância Sanitária*, Anvisa)^(^ 13 ^)^ to prevent and control bacterial resistance by 2020.

To answer the question, the objective was to analyze how, in the clinical practice of large hospitals in Minas Gerais, the adoption of prevention and control measures for the spread of bacterial resistance has occurred and propose a score that identifies this adoption among the institutions. It is expected, as a contribution, that defining an overview for this adherence by the institutions may come to subsidize the outline of the set of actions and public policies directed to the specific needs pointed out, as well as to identify gaps that need to be filled, seeking to consolidate the good practices in the assistance to the patient.

## Method

A cross-sectional study was conducted from February 2018 to April 2019 in 30 large hospitals in the state of Minas Gerais, after approval by the Ethics and Research Committee, under opinion No. 30783614.3.0000.5149. The institutions’ participation, after the consent of their manager, took place voluntarily and anonymously, without any financial benefit or coercion to participation. This study is part of the research entitled *Overview of the World Health Organization’s Global Challenges for Patient Safety in Large Hospitals in Minas Gerais*.

The population of this study was composed of large hospitals in the state of Minas Gerais, recognized as public, philanthropic, private or university hospitals, which provided services of medium to high complexity and accepted to participate in the research. For selecting the institutions, a survey on general hospitals was carried out, according to the National Registry of Health Establishments (*Cadastro Nacional de Estabelecimentos de Saúde*, CNES), identifying 542 hospital institutions as general hospitals, of which 32 were classified as large hospitals, that is, those who registered to have between 150 to 299 beds, in accordance with ordinance No. 2,224/GM^(^ 18 ^)^.

For the invitation to the institutions, the objectives of the research, its relevance and contributions were submitted through an invitation letter and telephone contact made by the State and Municipal Health Secretariats, highlighting the voluntary, non-gratified, confidential and secrecy character regarding the identity of the participants, risks and benefits.

After acceptance of the institution, given by the hospital manager, visits were scheduled, and the research team, during the visit to the institution, conducted face-to-face interviews with the hospital manager, the coordinator of the Hospital Infection Control Service (*Serviço de Controle de Infecção Hospitalar*, SCIH) and the care coordinators of the Inpatients Units (IUs) and the Intensive Care Center (ICC) to identify the characteristics and the sociodemographic profile of the hospital, to know the safety policies adopted for the prevention and control of bacterial resistance, to know the actions taken to contain bacterial resistance, and to evaluate the knowledge of the professionals on the measures to prevent the spread of antibiotic-resistant bacteria in the institutions.

These professionals were selected because they are responsible for implementing and promoting actions to contain bacterial resistance at the institutional level. A situational diagnosis was also carried out in the health care units (IUs and ICCs) to evaluate, in the practice, actions to control bacterial resistance. Data was collected in the same visit, following three simultaneous stages, described in Figure 1.

**Figure 1:** Stages for conducting the study

Stage	Data collection instrument	Method	Participants	Purposes
1^st^	Structured questionnaires	Face to face interview	Hospital Manager and Hospital Infection Control Service Coordinator*	To characterize the sociodemographic profile of the hospitals and the SCIH team, to know the patient safety policies adopted in the institutions, to describe HAIs surveillance and prevention actions, and to identify the existence of institutional standards and protocols related to the use of antibiotics, control of bacterial resistance and encouraging hand hygiene.
2ª	Structured questionnaires	Face to face interview	Coordinators of the Inpatient Unit and Intensive Care Unit^†^	To identify knowledge on the actions implemented and carried out on surveillance and prevention of HAIs, control of bacterial resistance and standard precautions and isolation measures.
3ª	Structured questionnaires	Note	Employees and physical area of the Inpatient and Intensive Care Units^‡^	To evaluate the conditions for the adoption of measures for the prevention of HAIs, control of bacterial resistance and hand hygiene in the clinical practice and to check the availability of infrastructure and supplies that guarantee prevention actions through a situational diagnosis.

* An interview was conducted with the Hospital Manager and with the SCIH Coordinator;

† An interview was conducted with the IU Coordinator and with the ICC Coordinator;

‡ A situational diagnosis was made in the IU and in the ICC

The data collection instruments were based on the guidelines proposed by the WHO for hand hygiene^(^ 19 ^)^ and in the guide of the Center for Disease Control (CDC) of Atlanta, 2010 (CLABSI Baseline Prevention Practices Assessment Tool For States Establishing Hai Prevention Collaboratives Using Arra Funds, which has been translated and adapted to the Brazilian reality)^(^ 20 ^)^.

For assessing the behaviors against bacterial resistance, the document for investigation and control of multi-resistant bacteria proposed by the Anvisa was adopted^(^ 21 ^)^. The instruments were previously submitted to content, criterion and construct validation in a pilot study, whose data were not included in the final analysis. The data collection instruments were composed of open and closed questions and are described below.

*Hospital Manager:* The data collection instrument consisted of a structured questionnaire, with the purpose of characterizing the sociodemographic profile of the hospitals and knowing the patient safety policies adopted at the institution.

*Hospital Infection Control Service Coordinator:* This is a semi-structured instrument, whose purpose was to characterize the hospital infection control service team, describe the surveillance and prevention actions of HAIs, and identify the existence of standards and protocols related to the use of antibiotics, control of bacterial resistance, prevention of HAIs and encouraging hand hygiene.

*Coordinators of the Inpatient and Intensive Care Units:* Through this structured questionnaire, composed of closed questions, the objective was to identify the knowledge of the coordinators of the care units on the actions implemented and performed on surveillance and prevention of HAIs, control of bacterial resistance and standard precautionary measures and isolation.

*Situational diagnosis in Inpatient and Intensive Care Units:* For the situational diagnosis, a structured questionnaire was used, composed of closed questions, with the objective of evaluating the adoption of measures for the prevention of HAIs, control of bacterial resistance and hand hygiene in the clinical practice of the professionals and also to evaluate the infrastructure and the provision of inputs that favor adherence to the good practices.

After this stage on identifying policies, knowledge and practices, the data were analyzed, and a score was developed based on guidelines considered as the gold standard for controlling the spread of bacterial resistance, such as the rational use of antibiotics, adherence to hand hygiene, and standard and contact precautions^(^ 22 ^)^. For constructing the ranking, scores were predicted, seeking to know and determine the degree of adherence to the measures and potential weaknesses in adhering to these guidelines^(^ 23 ^)^, as per Figure 2.

**Figure 2:** Proposal of the score for coping with bacterial resistance according to the global action proposed by the WHO

Item	Measurements	Score	Expected	Source
1	There is a protocol to guide the prescription of therapeutic antibiotics	1- Yes = 0.5 points 2- No/Does not know = 0.0 points	There is a protocol in the institution	Interview with the SCIH Coordinator
	There is a protocol to guide the prescription of prophylactic antibiotics	1- Yes = 0.5 points 2- No/Does not know = 0.0 points	There is a protocol in the institution	Interview with the SCIH Coordinator
2	Audits therapeutic antibiotics	1- Yes = 0.5 points 2- No/Does not know = 0.0 points	Audit of therapeutic antibiotics performed	Interview with the SCIH Coordinator
	Audits prophylactic antibiotics	1- Yes = 0.5 points 2- No/Does not know = 0.0 points	Audit of prophylactic antibiotics performed	Interview with the SCIH Coordinator
3	Knows the five moments for hand hygiene	1- Full answer: Before contact with the patient, before performing an aseptic procedure, after risk of exposure to body fluids, after contact with the patient, after contact with areas close to the patient = 1.0 point 2- Incomplete*/Does not know = 0.0 points	Knows completely the five moments recommended by the WHO for hand hygiene	Interview with the Care Units Coordinator
4(i)	Identifies the standard precautions	1- Full answer: Hand hygiene, using PPE when there is a risk for contact with blood or secretions and disposing sharps in appropriate containers = 1.0 point 2- Incomplete^†^/Does not know = 0.0 points	Completely identifies the standard precautionary measures	Interview with the Care Units Coordinator
5(i)	Personal protective equipment used when the patient is in contact precaution	1- Full answer: Procedure gloves and cloak = 1.0 point 2- Incomplete^‡^/Does not know = 0.0 points	Wears gloves and cloak when handling patient in contact precaution	Observation in Assistance Units
**Score Total**		**5 = completely adopts**		
		3 to 4.5 = partially adopts		
		2 to 2.5 = poor adoption		
		0 to 1.5 = does not adopt		

Incomplete: did not answer all five moments for hand hygiene;

† Incomplete: did not answer to all the measures considered in this study for standard precautions;

‡ Incomplete: did not use all the personal protective equipment required in this study when handling patients in contact precautions

The data obtained in the interviews and diagnoses were analyzed in the Statistical Package for the Social Sciences (SPSS), version 22.0, using descriptive statistics to characterize the studied population, by calculating absolute and relative frequencies.

## Results

From the interviews with the health managers, it was observed that the majority (70.0%; N=21) of the interviewees were female, and their main training was in the health area (70.0%; N=21). Also, by means of an interview with the health managers, the profile of the 30 participating institutions in Minas Gerais was identified. Of these, 43.3% (N=13) were located in the central region of the state, followed by the Southeast, with 20% (N=06), North and South, each with 10% (N=03). There was predominance of non-accredited hospitals, 63.3% (N=19). Of these, 60% (N=18) focused on teaching and research, which met high-medium complexity, and 43.3% (N=13) were philanthropic.

The accredited hospitals accounted for 36.7% (N=11) of the sample. Among the certifiers, 72.7% (N=08) were certified by the National Accreditation Organization (*Organização Nacional de Acreditação*, ONA); 9.1% (N=01) by the International Organization for Standardization (ISO) 9001; 9.1% (N=01) by Commitment to Hospital Quality (*Compromisso com a Qualidade Hospitalar*, CQH) and 9.1% (N=01) by the Canadian accreditation, from the Canadian Council on Health Services.

Regarding the accreditation level, 75% (N=06) of the institutions accredited by the ONA corresponded to level three, 12.5% (N=01) to level two and 12.5% (N=01) did not report. The hospitals accredited by the Canadian certifier and by the CQH presented diamond level and level one, respectively. The majority of the accredited hospitals, 45.4% (N=05), were in the Central Region of the state, followed by 36.4% (N=04) located in the Southeast Region, 9.1% (N=01) in the Region North, and 9.1% (N=01) in the South Region.

It was observed that the mean number of beds found in the study was 288 (153 - 1080), and 41 (9 - 155) intensive care beds. Among the ICUs types, the following prevailed: 100% (N=30) of the institutions had beds for adult patients, 60% (N=18) for neonatal patients, and 53.3% (N=16) for children/Pediatrics.

Considering the interviews with the coordinators of the SCIHs, 100% (N=30) were health professionals, 83.3% (N=25) nurses, 13.3% (N=04) physicians, and 3.3% (N=01) pharmacists. The mean number of professionals working in the SCIH was two (1-13) nurses, two (0-4) physicians, one (0-3) employee with administrative function, one (0-5) Nursing student, and one (0- 1) medical student.

It was also identified that all the services implemented actions to control the transmission of antibiotic-resistant bacteria in situations where the patient had colonization or infection associated with resistant bacteria. Among the actions mentioned, 93.3% (N=28) adopted contact precautions for patients with resistant bacteria, 60% (N=18) identified the beds, 56.7% (N=17) had private rooms, and 30% (N=19) individualized articles used in care, such as thermometers, stethoscopes and sphygmomanometers. In addition to these, 93.3% (N=28) of the coordinators of the SCIHs stated that they made technical visits in the sectors, with 46.7% (N=28) stating that they perform them at least annually.

In 93.3% (N=28) of the institutions, pre-established routines or protocols for the rational use of antibiotic prophylaxis in surgeries were referred by the services, and 86.7% (N=26) stated that they conducted audits. In 81.5% (N=22) of the institutions, the audit was performed by the physician, and 73.1% (N=19) conducted them daily. In the other institutions, the pharmacist or nurse carried out audits on a weekly, monthly or quarterly basis. Regarding therapeutic antibiotics, 86.7% (N=26) of the hospitals stated that they had protocols, and 83.3% (N=25) reported conducting audits. Most of the audits, 83.3%, (N=25), were performed by the physician on a daily basis. In a hospital, the audit was performed by a pharmacist weekly or quarterly.

The majority, 76.7% (N=23), of the SCIHs of the participating institutions carried out campaigns to encourage hand hygiene at least annually, and 93.3% (N=28) of the services provided training for the multi-professional team. Regarding training periodicity, 40% (N=12) performed it less than annually and 36.7% (N=11), annually.

The indicators on the adherence to hand hygiene were found in 93.3% (N=28) of the institutions, with 82.1% (N=23) consuming products such as soap and alcohol, 50% (N=14) direct observation, and 7.1% (N=02) indirect observation. In some institutions, using more than one method of monitoring adherence has been reported.

Through an interview with the coordinators of the assistance units (N=60), who were nurses in their entirety, their knowledge of the standard precautions was questioned, and 100% (N=60) stated that they knew about that precaution. However, when asked to cite the measures that compose it, 93.3% (N=28) of the interviewees in the ICUs and 86.7% (N=26) in the IUs were unaware of or answered incompletely about hand hygiene, use of personal protective equipment (PPE), and disposal of sharps in an appropriate container.

Regarding the five moments for hand hygiene, recommended by the WHO, the majority, 98.3% (N=59), of the nurses from the health care units reported knowing. However, when asked to describe the moments, 93.3% (N=28) of the interviewees of the IUs and 63.4% (N=19) of the ICUs were unaware of them or answered incompletely. The least remembered moments were the following: after contact with surfaces close to the patient and after exposure to body fluids.

During the situational diagnosis of the IUs (N=30) and ICUs (N=30), it was observed, among the professionals working in the clinical practice, which personal protective equipment was mandatorily used in the care of patients in contact precautions. The majority, 93.4% (N=56), of the professionals used the two pieces of equipment, the measure having the highest percentage of correct answers.

In the set of measures proposed for the construction of the score, analyzing the knowledge of the professionals from the inpatient and intensive care units interviewed at the institution, based on the answers obtained for the adoption of measures for the prevention and containment of bacterial resistance, it was observed that 90% (N=54) of the respondents in the care units did not completely identify the standard precautionary measures, the least mentioned being the disposal of sharps in appropriate containers, and 78.3% (N=47) did not fully know the five moments for hand hygiene, as shown in Figure 2.

For handling the patients in contact precautions, 93.4% (N=56) of the professionals used gloves and cloaks, 90% (N=27) of the institutions had protocols to guide the prescription of antibiotics, and 85% (N=26) conducted audits.

As a result of the observational diagnosis, the position of the soap and alcohol dispensers was verified. It was evident that both were side by side in 58.3% (N=35) of the Nursing posts of the care units visited, the majority of which, 63.3% (N=19), was located in the IUs, and 53.3% (N=16) in the ICUs. These were also side by side in 36.6% (N=22) of the patients’ rooms, 35.0% (N=21) of the expurgations, and 23.3% (N=14) of the corridors. It was observed that, in 96.7% (N=58) of the health care units, the health professionals did not have alcohol gel in pocket bags available for individual use.

**Figure 3:**
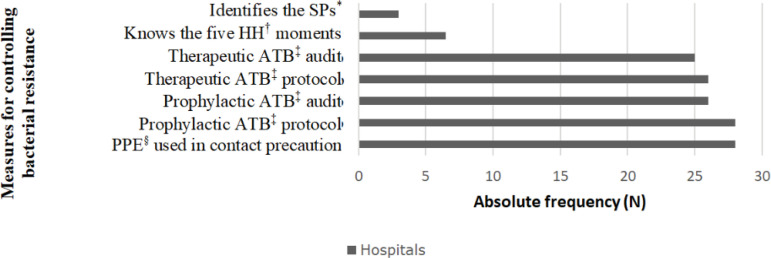
Distribution of the adoption of the measures proposed for the score, according to their adherence among the large hospitals of Minas Gerais (n=30), participants of the study. Belo Horizonte, MG, Brazil, 2019 ^*^SP = Standard precaution; ^†^HH = Hand hygiene; ^‡^ATB = Antibiotic; ^§^PPE = Personal protective equipment

**Figure 4:**
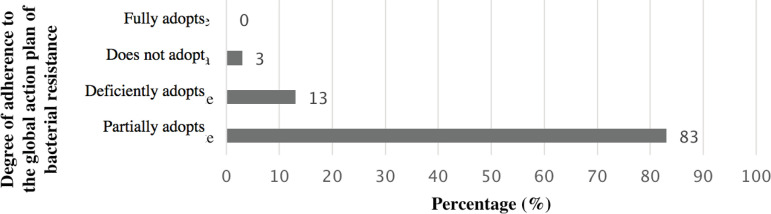
Adherence of the institutions participating in the study to the score of the WHO global bacterial resistance action plan among the large hospitals of Minas Gerais (n=30). Belo Horizonte, MG, Brazil, 2019

Based on the measures selected for the composition of the score (protocols to guide the prescription of prophylactic and therapeutic antibiotics, perform antibiotic audit, know the five moments for hand hygiene, identify the standard precautions, and correctly use gloves and cloaks when handling patients in contact precaution), there was a difference in adherence to these measures on the part of the hospitals, as shown in Figure 3. From the adherence or not to these measures, each hospital participating in the study was assigned a score, as shown in Figure 4.

## Discussion

The majority, 43.3% (N=13), of the large general hospitals were located in the central region of the state and were philanthropic and non-accredited entities. The concentration of hospitals in this region confirms the uneven distribution of health services in Minas Gerais, demonstrating that the most economically developed territories bring together medium-high complexity services, becoming a reference for other regions^(^ 24 ^)^.

This finding is similar to the national situation, where there is greater concentration of hospitals in the Southeast or Central Regions, indicating that the higher the socioeconomic status of the individuals or regions, the better the health status and access to the health services^(^ 25 ^)^.

Concerning the financing entity, difficulties related to the scarcity of resources, rising costs of care, technological advances, and the need to improve the quality of care provided to patients were mentioned^(^ 26 ^)^. Philanthropy has an important part of the Brazilian hospital park, with a special presence among healthcare service providers for the Unified Health System (*Sistema* Único *de Saúde*, SUS)^(^ 26 ^)^.

Non-accredited institutions were prevalent in this study, which is in line with data from the ONA, which revealed 43 accredited hospitals in the state of Minas Gerais^(^ 27 ^)^. National or international accreditation processes that evaluate and certify health services in terms of meeting patient care requirements contribute to improving safety, process quality, and continuous improvement^(^ 28 ^-^ 30 ^)^.

The literature argues that accredited institutions invest in the implementation of processes and policies to promote improvement and adherence to good practices, as accreditation is able to promote changes in hospital management and decision-making processes, in addition to stimulating the commitment of the hospital with the quality and patient safety assessment processes^(^ 28 ^-^ 30 ^)^.

Recognizing the worldwide impact of bacterial resistance on public health, the WHO has considered its containment as a global priority. Five areas of action have been defined for its control: improving awareness and understanding on bacterial resistance through communication, education and training; regulation and rational use of antibiotics; encouraging research for the development of new antimicrobials; improvements in the surveillance systems for infections associated with resistant pathogens and promotion of effective measures to reduce the transmission of these pathogens to susceptible individuals in the health services^(^ 4 ^)^.

In this study, the existence of protocols to guide the prescription of antimicrobials and to carry out audits was considered, as well as complete knowledge by the health professionals on the five moments for hand hygiene, the identification of the standard precautions by the professionals and of the individual protective equipment for mandatory use for patients in contact isolation, as the necessary measures for the control of bacterial resistance in large hospitals in Minas Gerais. Rational use of antimicrobials, improvements in adherence to hand hygiene, and standard and contact precautions are referred to in the scientific literature as gold standard measures for the control of bacterial resistance^(^ 22 ^,^ 31 ^)^.

In most of the institutions participating in the study, the existence of protocols to guide the prescription of antibiotics and of audits carried out by the SCIH medical team was mentioned. The standardization of antimicrobials is linked to a drug control policy and programs for the rational use of these drugs, representing extremely important actions in the optimization of antimicrobial therapies and in minimizing the occurrence of bacterial resistance^(^ 32 ^-^ 33 ^)^.

A number of studies indicate that protocols developed based on local microbiology have a direct impact on reducing infections and colonization by resistant bacteria in hospitalized patients^(^ 33 ^-^ 34 ^)^. In addition, it is emphasized that these protocols focus on continuing education, provide information feedback, and measure the results by means of indicators of adherence to good practices and actual consumption of antimicrobials by care unit^(^ 35 ^-^ 37 ^)^.

The knowledge on the five moments for hand hygiene was considered the second item for containing bacterial resistance in the health services; however, it was one of the measures with the lowest percentage of correct answers. In line with the results found, a study carried out in a Brazilian hospital verified that 56.7% of the professionals claimed to know the five moments for hand hygiene; however, 8.1% correctly described the moments, and just over 50% reported having received hand hygiene training^(^ 38 ^)^. In two hospitals of Paraná, the knowledge of Nursing professionals on hand hygiene was assessed and it was concluded that 86.5% of the interviewees did not fully know the five moments^(^ 39 ^)^.

These findings confirm that, although the professionals recognize hand hygiene as one of the essential measures to control the spread of resistant microorganisms in the hospital environment, knowledge on the five moments remains a challenge^(^ 38 ^-^ 40 ^)^. Above all, the different opportunities for adhering to the care of the same patient.

The moments after contact with surfaces close to the patient and after exposure to body fluids were the indications most overlooked by the professionals. Differently from the current study, a research study that evaluated the opportunities for hand hygiene by health professionals demonstrated that the opportunities before contact with the patient and before the aseptic procedure were the ones with the lowest adherence^(^ 41 ^)^. This reinforces that the lack of knowledge on the hand hygiene moments may come to impact the adherence to opportunities in the clinical practice since, in the present study, the professionals did not recognize the relevant moments for the risk of transmitting antibiotic-resistant bacteria.

Regarding the identification of the standard precautions, it was verified that most of the professionals interviewed did not report hand hygiene and the disposal of sharps in an appropriate container as the measures that comprise them. The literature shows that knowledge on the standard precautionary measures is lower than desired, demonstrating that the professional does not have adequate knowledge on this important principle^(^ 42 ^)^.

A study conducted in Europe revealed that 21% of the professionals are unaware of hand hygiene as an indication of the standard precaution^(^ 43 ^)^. The importance of the professionals’ knowledge in adhering to the recommendations and the relevance of training to change the reality found is reinforced^(^ 44 ^)^.

With regard to the mandatory PPE in the care of patients in contact precautions, in this study, the use of cloaks and gloves by the professionals in most of the hospitals was evidenced. The national and international recommendations reinforce that contact precautions should be initiated from proof of colonization/infection by resistant bacteria^(^ 22 ^,^ 45 ^)^.

A study that evaluated the impact of implementing contact precautions for all patients in a burn unit after an outbreak of *Acinetobacter baumanii* demonstrated that the application of contact precautions for all the patients in an ICU may not reduce colonization by resistant microorganisms among the patients^(^ 46 ^)^. It was verified that the reduction in the spread of resistant microorganisms among the patients is due to multifaceted strategies, which involve the rational use of antibiotics, hand hygiene, and adherence to the standard and contact precautions^(^ 45 ^)^.

However, a study that evaluated the adoption of contact precautions, before confirmation of colonization or infection by resistant microorganisms, highlighted the importance of surveillance cultures in the tracking of patients in an ICU and the implementation of contact precautions for all the patients^(^ 47 ^)^.

It is also emphasized that the identification of the colonized/infected patient before contact isolation is necessary^(^ 45 ^)^, the importance is reinforced that the contact precaution is implemented for all the colonized patients and that it remains until the end of the hospitalization, according to the recommendations of the Guideline for Isolation Precautions: Preventing Transmission of Infectious Agents in Healthcare Setting^(^ 22 ^)^.

Despite the high adherence to the PPE to provide assistance to patients in contact precautions, in the present study, communication failures, work overload, inadequate physical structure, inaccessibility to protective equipment, and organizational and managerial aspects were referred to as factors that interfere with the use of personal protective equipment by the professionals^(^ 48 ^)^.

For the proposal of the score, in the group of hospitals evaluated it was verified that the measures of prevention and/or containment of bacterial resistance were not fully adopted in the clinical practice. As evidenced during the interviews carried out in the health care units, insufficient knowledge on the standard precautions and the five moments for hand hygiene stood out.

The partial adherence of the hospitals to measures to prevent bacterial resistance is related to the prevention and control policy for antibiotic-resistant bacteria developed by the hospitals, since it was reported as the focus of action in only one hospital; the professionals’ lack of knowledge on preventive measures such as the standard precautions and the five moments for hand hygiene, which, in turn, lead to inconsistent conducts in the clinical practice, favoring the spread and lack of control of bacterial resistance^(^ 38 ^,^ 44 ^)^.

Regarding the policy of control of bacterial resistance developed, it is related to the frequency of epidemiological surveillance actions and monitoring of indicators associated with resistant bacteria carried out by the SCIHs in the hospitals. In this sense, the role of the SCIH technical visits to the assistance sectors is highlighted.

A number of studies have shown that the presence of a SCIH professional in the units favors adherence to infection prevention measures and is an opportune moment for guidance and identification of gaps^(^ 49 ^-^ 50 ^)^. The view of specialist professionals with a focus on infection prevention and control tends to favor improvements in the work and patient care processes, in addition to providing the approach and guidance of the professionals in the clinical practice^(^ 49 ^-^ 50 ^)^.

Regarding the use of indicators, it was observed that most of the hospitals evaluated the occurrence of HAIs associated with resistant bacteria. Monitoring indicators related to the adherence to the standard precautionary and contact measures, in addition to hand hygiene (direct/indirect observation/product consumption) are important instruments for measuring the adherence of the professionals in the clinical practice to bacterial-resistance control measures, in addition to enabling identification of gaps^(^ 51 ^)^.

The professionals’ lack of knowledge on the preventive measures has a direct impact on the clinical practice because, when the professionals are unaware of aspects that include the ways of transmissibility of resistant bacteria and of the prevention measures, they tend to underestimate the risks and not to adopt such measures in the clinical practice^(^ 38 ^,^ 44 ^)^. In this sense, it is essential to carry out institutional training, which results in improving the knowledge of the multidisciplinary team, care processes and activities. The trainings should promote the development of new skills in care, integrating all the professional categories^(^ 51 ^)^.

Finally, the inadequate infrastructure, associated with the provision of soap and alcohol at the points of assistance, are also important for the containment of bacterial resistance. The side-by-side arrangement of the dispensers in the Nursing stations, evidenced in the present study, can implicitly reinforce the sequential procedure, a practice that should not be adopted by the team^(^ 7 ^,^ 52 ^-^ 53 ^)^.

In addition, a number of studies reinforced the importance of the existence of alcohol dispensers at the points of assistance, as recommended by Resolution RDC No. 42, 2010^(^ 54 ^)^, as well as booklets that remind the professional to perform hand hygiene, availability of good quality personal protective equipment and in sufficient numbers to favor adherence to hand hygiene and to the standard and contact precautionary measures^(^ 7 ^,^ 55 ^)^.

Containing bacterial resistance is a WHO goal and, in Brazil, the Anvisa has published several documents in line with these recommendations^(^ 4 ^,^ 13 ^)^. However, the greatest difficulty is related to the implementation of these guidelines in the clinical practice, as evidenced in this study.

Controlling bacterial resistance is a major challenge for health institutions, especially the Brazilian. So that, in 2016, the National Program for the Prevention and Control of Infections Related to Health Care (*Programa Nacional de Prevenção e Controle de Infecções Relacionadas à Assistência à Saúde*, PNPCIRAS) for the 2016-2020 five-year period was published, among whose objectives is to prevent and control the spread of microbial resistance in the health services^(^ 56 ^)^.

Aiming at its consolidation, national events focusing on inducing infection prevention and control actions for managers, health surveillance technicians and coordinators of infection control commissions have been promoted. In addition, the states were encouraged to implement HAI prevention and control programs, national guidelines for the preparation of the Antimicrobial Use Management Program in Health Services were published, and proposals for national HAI prevention and control actions were submitted to the Ministry of Health (*Ministério da Saúde*, MS)^(^ 56 ^)^.

Despite these advances, in 2020 the program is concluded and, however, it appears that much still needs to be done with regard to the control of bacterial resistance in Brazil and worldwide. So much so that projections point to a considerable human and financial cost related to bacterial resistance, indicating that, if it is not controlled by 2050, it will be responsible for the death of ten million more people each year and for a reduction between 2.0% and 3.5% of the countries’ gross domestic product (GDP), which will cost the world more than 100 trillion dollars^(^ 8 ^)^.

As for the limitations, it can be pointed out that the number of beds informed in the national registry of health institutions by the IT department of the Unified Health System (DATASUS) was out of date. During the research, it was noticed that the number of beds informed by the managers was different from that indicated in the register. In view of this limitation, the number of beds informed by the managers at the time of the interview was considered. In addition, the difficulty of agreement of other institutions to carry out the research stands out, which prevented a larger sample.

Although the observational model is considered an important strategy in the analysis of processes and routines, the Hawthorne Effect may have occurred. Although the professionals were followed up in a way that they did not realize that they were being observed and according to the opportunity of the action performed, they were aware of the presence of the researchers; therefore, an increase in the adherence to some practices may have happened. To minimize this effect, observations were made concurrently with the situational diagnosis.

In addition, despite being a representative sample, the care units’ observations were made with reference to the hospital unit. However, the results found have been compatible and can be generalized, since that the considered sample unit was large hospitals in Minas Gerais, and more than 90% of these institutions participated in the study.

Although in this study it was not possible to observe all the measures for the prevention of bacterial resistance in the clinical practice, such as the prescription of antimicrobials and the conduction of audits, the present research allowed the knowledge of a local reality, in need of attention, review and improvement by means of infection control policies.

The findings of the present study point to a reality that certainly extrapolates a setting for the state of Minas Gerais, which can be comparable and representative of the national context, considering the findings of different studies conducted in other regions of the country.

In this sense, it is reinforced that the adherence gaps can express that, although the Anvisa regulations and recommendations are being published, in the reality of the institutions the problem is still very serious and exists for different reasons, such as those found in this study: lack of the professionals’ knowledge on the five moments for hand hygiene and standard precautions, inadequate logistics of soap and alcohol dispensers at the points of assistance, and absence of specific policies and with regulatory power to control bacterial resistance.

Despite the limitations, this study contributed to identify the actions and measures to control bacterial resistance developed in hospitals and, mainly, those that needed more attention. It brought the reality of institutional policies and practices closer by the involvement of managers, the infection control commission and, above all, by revealing the knowledge of the frontline professionals directly involved in the assistance. Thus, undoubtedly, the results found in the present study can be extrapolated, revealing aspects that must be investigated and remedied throughout the country, supporting policies and practices for the different regions.

## Conclusion

Regarding the prevention and control actions carried out by the hospitals, it was noticed that most of them monitored adherence to hand hygiene, had protocols and conducted antibiotic audits, implemented standard and contact precautions, identified the bed of the patient with resistant bacteria, and adopted routine surveillance cultures.

When analyzing the measures that constituted the score, it was evidenced that most of the hospitals participating in the study partially adopted the measures for the prevention and control of bacterial resistance. Despite the existence of prophylactic and therapeutic antibiotic protocols, the performance of their audits and the adherence to the use of personal protective equipment when assisting a patient in contact precautions, the standard precaution identification measurements and knowledge on the five moments for hand hygiene have not been fully answered.

Regarding the guidelines chosen for the proposal of the score, among which, rational use of antimicrobials and improvements in the adherence to hand hygiene and to the standard and contact precautions, are referred to in the scientific literature as gold standard measures for the control of bacterial resistance. These should compose a multi-modal strategy within the institutions. Despite being recognized for resistance control, it was observed that they are not fully adopted in the clinical practice, which may be related to the professionals’ lack of knowledge and to inadequate infrastructure.

The lack of knowledge of the Nursing professionals on the five moments for hand hygiene and on the standard precautions, inadequate logistics of soap and alcohol dispensers at the points of assistance, and the absence of a specific policy with regulatory power to control bacterial resistance represent gaps for the adherence to the actions to prevent and control bacterial resistance in large hospitals in the state of Minas Gerais.

The state of Minas Gerais is the largest in number of Patient Safety Centers, so it is necessary to investigate how patient safety actions and policies are being conducted in the hospitals. It is suggested that similar studies be conducted in other states to define a national overview.

The need to consolidate the patient safety policies in the health institutions and the involvement of senior management to carry out actions in the clinical practice is highlighted. In addition, measures such as the identification of the standard precautions and the knowledge of the five moments for hand hygiene need to be reviewed among the health professionals, mainly in Nursing, the professional category that is most present during health care, demonstrating the importance of continuing education in an attempt to increase adherence to these practices and as a tool capable of influencing patient safety actions and containing the spread of resistant bacteria.
